# Hybrids and mini-cutting: a powerful combination that has revolutionized the *Eucalyptus* clonal forestry

**DOI:** 10.1186/1753-6561-5-S7-I18

**Published:** 2011-09-13

**Authors:** Teotônio Francisco Assis

**Affiliations:** 1 ASSISTECH LTDA

## Background

Eucalypt canker disease (*Chrysoporthe cubensis*) was the main driver of the concept of eucalypt clonal forestry in Brazil. Initially clonal forestry was based on outstanding spontaneous hybrids resistant to canker disease. Since the recognition that cloning superior inter-specific hybrids could be an important general strategy, several crosses were made for many different purposes, especially pulp & paper, charcoal and more recently veneer and solid wood. This strategy has been responsible for a significant advance in the forest productivity of *Eucalyptus*. The underlying foundation for this strategy has been the exploitation of heterosis observed in most inter-specific crosses.

Due to its importance, hybridization and cloning currently constitute a key component of almost all forest-based industrial plantations in Brazil. Significant progress has taken place in the last few years with the different techniques used to carry out controlled crosses and cloning.

## Controlled crosses

The fact that *Eucalyptus* flowers are hermaphrodite and protandreous makes the traditional method of controlled crosses difficult to perform. This method is based on the exploitation of protandry, which involves emasculation and isolation, prior to pollination. It needs several visits resulting in a technique of low operational yield.

After discovering that different treatments in stile and stigma during anthesis could immediately induce flower receptivity [3], it was developed the OSP (One Stop Pollination) technique [4]. Later it was found that this receptiveness could be achieved just before anthesis [5]. Combining these two discoveries a new technique was developed [2], called AIP (Artificially Induced Protogyny). This technique consists of the artificial "transformation" of protandry into protogyny, obtained by cutting the top of the floral bud operculum together with the upper third of the stile. This is done during the pre-anthesis stage, i.e., when the flower is still closed. Pollen can be applied immediately after induction. Additionally the concept of indoor breeding orchards was developed, precluding the need to isolate individual flowers, umbels or branches, enabling the isolation of whole plants in a collective way.

The combined use of these technologies (AIP and indoor breeding orchards) currently allows large scale controlled crosses, and enables the production of highly superior full-sib families, a task formerly considered technically and economically difficult in *Eucalyptus*. Operational productivity improved from 35 to 400 pollinated flowers/person/hour.

## Cloning techniques

Cloning techniques have been significantly enhanced in the last years. The first major leap occurred in 1992, when micro-cutting was invented [1] and later mini-cutting. The micro-cutting is a rooting method, where the propagules are obtained exclusively from shoot apices, originated from micro-propagated plants. Mini-cuttings, on the other hand, come from axillary sprouts of rooted stem-cuttings. After the establishment of the mini-clonal hedges, the two techniques are identical, varying only in the origin of the initial source of propagules. Micro-cutting and mini-cutting are the most modern concepts for large scale cloning of *Eucalyptus* species. They are implemented using hydroponic systems such as sand bed with drip irrigation or intermittent flooding with very specific nutritional parameters that allow commercial scale production of sprouts. Although significant progress has been made in these robust and functional systems, new approaches to clone *Eucalyptus* species are opening important opportunities to improve operational results and also contemplating recalcitrant species in the general hybridization & cloning strategy. One of these new approaches is cloning selected trees from lignotuber tissue. Tested originally at Arcelor Mittal Bioflorestas, cloning from lignotubers constitute a new advance to be soon incorporated in the operational procedures. Another promising approach is to cover mini-stumps with mini-tunnels. Preliminary results of this method at Aperan Bioenergia have shown that productivity, rooting rate and root quality are improved, reducing the formation of callus.

After more than thirty years of experience on deriving clones from inter-specific hybrids, this general strategy, together with the evolution of silvicultural practices, have been the major drivers of the progress experienced in forest productivity in Brazil. The evolution of pulp productivity obtained by cloning superior hybrids has been impressive. While in 1970 the average MAI-Cellulose was 6 ton/ha/year it now reaches 12 ton/ha/year. New projections now work with the possibility of achieving a potential productivity of about 16 ton/ha/year by 2015. One of the key elements for this further jump in productivity is the rapidly increasing use *E. globulus* in hybrid breeding programs. *E. globulus* has superior wood qualities such as low lignin content and high S/G ratio (one of the highest in woody plants) that result in easier cooking, low specific consumption (high wood density and pulp yield) and good bleaching ability. The use of adapted species and clones in crosses with *E.globulus* is yielding a new breed of elite hybrid clones for subtropical and tropical areas.
